# A Split G-Quadruplex and Graphene Oxide-Based Low-Background Platform for Fluorescence Authentication of *Pseudostellaria heterophylla*

**DOI:** 10.3390/s141222971

**Published:** 2014-12-03

**Authors:** Zhenzhu Zheng, Juan Hu, Zhaodong He

**Affiliations:** 1 Institute of Drug Research, Fujian Academy of Traditional Chinese Medicine, Fuzhou 350003, China; E-Mails: smilepearl@126.com (Z.Z.); Zhaodong@gmail.com (Z.H.); 2 Fujian Quanzhou Children's Hospital, Quanzhou 362000, China; 3 The College of Pharmacy, Fujian University of Traditional Chinese Medicine, Fuzhou 350122, China

**Keywords:** split G-quadruplex, graphene oxide, fluorescence, *Pseudostellaria heterophylla*

## Abstract

A label-free split G-quadruplex and graphene oxide (GO)-based fluorescence platform has been designed to distinguish *Pseudostellaria heterophylla* (PH) from its adulterants based on the differences in their nrDNA ITS sequences. Herein, GO has been first introduced to capture G-rich probes with 2:2 split mode and then decrease the background signal. As T-DNA exists, the probes leave the GO surface to form double-stranded structures followed by the formation of the overhanging G-rich sequence into a G-quadruplex structure, which combines quinaldine red specifically to produce a strong fluorescence signal. In addition, this strategy allows detection of T-DNA in a wide range of concentrations from 1.0 × 10^−8^ to 2.0 × 10^−6^ mol·L^−1^ with a detection limit of 7.8 × 10^−9^ mol·L^−1^. We hope that the split G-quadruplex/GO platform can be utilized to further develop gene identification sensors in Traditional Chinese Medicine or other analysis areas.

## 1. Introduction

Recently, Traditional Chinese Medicine (TCM) is facing serious evaluation and regulation challenges [1]. In particular, adulterated medicinal herbs which may have weaker or completely different pharmacological actions [2] are still commonly misused in the market due to their similar morphological features or lower cost [3]. *Pseudostellaria heterophylla* (PH), possessing similar but milder efficacy than ginseng, has been commonly used to treat night sweats, asthenia after illnesses, lung and spleen diseases for children [4–6]. However, there exist many adulterants in the market, for example, *Lophatherum gracile* (LG), *Liriope platyphylla* (LP), *Ophiopogon japonicus* (OJ), *Stemona sessilifolia* (SS) and *Stemona japonica* (SJ) [7]. Based on well-characterized marker compounds, scientists have developed various authentication methods, e.g., HPLC-MS [8], HPCE [9], GC-MS [10] and NIR spectroscopy [6]. Furthermore, nuclear ribosomal DNA internal transcribed spacer (nrDNA ITS) possessing highly conserved and variable regions [11] has been frequently used as genetic marker for the authenticity of Chinese herbal medicines [12]. The common techniques for genetic authentication include RAPD [13], RFLP [14] and assembly-free genome sequencing [15]. However, the practicality of these methods is limited by their reliability, sensitivity and cost of execution. Therefore, there is an urgently need to develop a reliable, sensitive and low-cost authentication method.

G-quadruplexes, which are formed by the fold-over of G-rich sequences [16], can accommodate fluorescent ligands specifically through π-stacking, loop or groove recognition, and this affects the fluorescence signal [17]. For example, quinaldine red is reported to have a weak fluorescence signal itself and display strong enhancement upon binding G-quadruplexes [18,19]. Furthermore, much research has been done on splitting the unusual G-rich sequence into two equal parts (2:2 split mode) for the sake of extraordinary selectivity and a more flexible assay design. However, the 2:2 split parts could easily assemble into a G-quadruplex in the absence of target DNA and then produce a background signal [20].

In recent years, the novel nanomaterial graphene oxide (GO) has attracted more and more attention. In particular, GO with epoxides, carboxylic acids and hydroxyl groups can be obtained through acid exfoliation of graphene [21], and these active groups render GO water-soluble and suitable for biosensors/bioassays [22]. Moreover, it is reported that GO can capture single-stranded DNA strongly through π-stacking interactions [23], yet capture rigid double-stranded DNA weakly due to the nucleobases within the negatively charged phosphate backbone [24]. G-quadruplexes are also reported to have weak affinity for GO due to their high space charge density [25]. This interesting property of GO of differentiating between diverse DNA structures has been utilized for DNA biosensors [26].

In this study, we construct a fluorescence platform for distinguishing PH from its adulterants based on their different ITS sequences. Herein, G-quadruplex with 2:2 split mode is applied in the design of the probe and a GO nanosheet to adsorb the probe and decrease the background is introduced. In addition, quinaldine red is chosen as fluorescent indicator for revealing G-quadruplex formation.

## 2. Experimental Section

### 2.1. Materials

GO dispersion (1 g·L^−1^) was obtained from Xianfeng Nanotechnologies Co., Ltd. (Nanjing, China). Quinaldine red was purchased from Aladdin Chemistry Co., Ltd. (Shanghai, China). All oligomers were synthesized by Sangon Biotechnology Co., Ltd. (Shanghai, China). Tris-HCl buffer (pH 7.0) contains 25 mM Tris, working buffer (pH 8.0) contains 50 mM Tris and 100 mM KCl. The oligomers (5.0 × 10^−5^ mol·L^−1^) were prepared in Tris-HCl buffer and quinaldine red solution (5.0 × 10^−4^ mol·L^−1^) was prepared in the working buffer.

### 2.2. Assay Procedure

In the split G-quadruplex/GO assay, probe (0.5 μL, 5.0 × 10^−5^ mol·L^−1^) was first added to GO dispersion (2.5 μL, 1 g·L^−1^) for 60 min. Second, a certain volume of T-DNA (5.0 × 10^−5^ mol·L^−1^) and working buffer (25 μL) were added, and this uniform mixture was incubated for 60 min. Subsequently, working buffer along with quinaldine red (5 μL, 5.0 × 10^−4^ mol·L^−1^) was added to adjust the total volume to 250 μL. After 60 min, the fluorescent spectrum was scanned on a fluorescence spectrophotometer (Varian Cary Eclipse, Palo Alto, CA, USA). The instrument parameters were set as follows: λ*_ex_* = 562 nm (slit 10 nm), λ*_em_* = 580–800 nm (slit 10 nm). In the circular dichroism (CD) experiments, the final concentrations of probes and T-DNA were 5.0 × 10^−6^ mol·L^−1^ and which of GO was 10 mg·L^−1^. CD spectra were measured on a circular dichroism spectrometer (Aviv Model 420, Lakewood, NJ, USA) at room temperature. The wavelengths were scanned from 220 to 320 nm, with a step of 2 nm and bandwidth of 1 nm.

## 3. Results and Discussion

### 3.1. Fluorescence Property of Quinaldine Red

It has been reported that the fluorescence intensity of quinaldine red increases greatly in the presence of G-quadruplexes [18]. Furthermore, its fluorescence performance in the presence of different DNA conformations (in Table 1) has been investigated.

**Figure 1.**
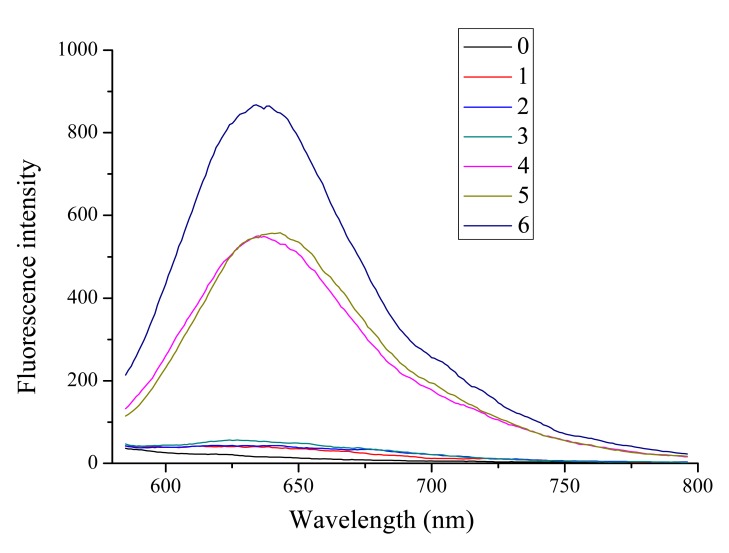
Fluorescence spectra of quinaldine red. Curve 0, quinaldine red (4 × 10^−6^ mol·L^−1^); Curves 1–6, quinaldine red (4 × 10^−6^ mol·L^−1^) + corresponding oligomer (4 × 10^−6^ mol·L^−1^).

As indicated in Figure 1, the fluorescence of quinaldine red itself is quite low (curve 0) and then increases little in the presence of oligomers **1, 2** (dsDNA; curves 1, 2) and oligomer **3** (tetramer-type G-quadruplex, curve 3). Moreover, the fluorescence increases strongly in the presence of oligomer **4** (hairpin-type G-quadruplex, curve 4) and oligomer **5** (G-quadruplex, curve 5). Finally, it reaches a maximum in the presence of oligomer **6** (parallel G-quadruplex, curve 6). Therefore, oligomer **6** is utilized in the probe design.

### 3.2. Probe Design and Optimization

According to reports, there exists certain variation in the nrDNA ITS region between PH and its counterfeit species [7]. As shown in Table 2, a section of ITS sequence specific for PH is labeled as T-DNA and works as target, while sequences specific for the adulterants are labeled as C-DNA.

**Figure 2.**
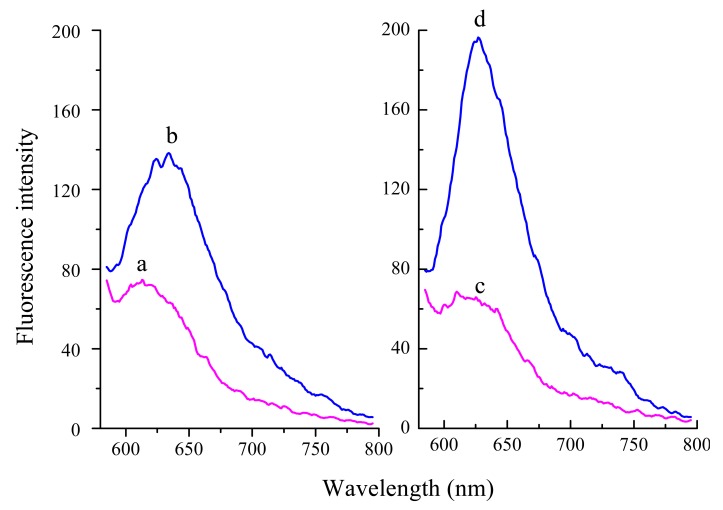
Fluorescence spectra of (**a**) GO + probe 1; (**b**) GO + probe 1 + T-DNA; (**c**) GO + probe 2; (**d**) GO + probe 2 + T-DNA.

Furthermore, based on T-DNA and oligomer 6, two types of probe have been designed: probe 1 and probe 2 (probe 2-a, probe 2-b). Firstly, the G sequence of PS2.M at the 5′-terminus is moved to the 3′-terminus. Probe 1 possesses one section from the 3′-terminus complementary to T-DNA and the rest is PS2.M sequence. According to the equal 2:2 split mode, probes 2-a and -b both have one section complementary to different regions of T-DNA and the other section being two GGG repeats, respectively. Herein, the performance of probe 1 and probe 2 has been investigated and compared under the same conditions. In the absence of T-DNA, probe 1 system displays a little higher fluorescence signal (Figure 2, curve a) compared with probe 2 (curve c). Moreover, in the presence of T-DNA, probe 2 system (curve d) exhibits an obviously higher fluorescence signal than probe 1 system (curve b). Therefore, probe 2 with 2:2 split mode is chosen in the following experiments.

### 3.3. Principle of the Split G-Quadruplex/GO Platform

**Scheme 1.**
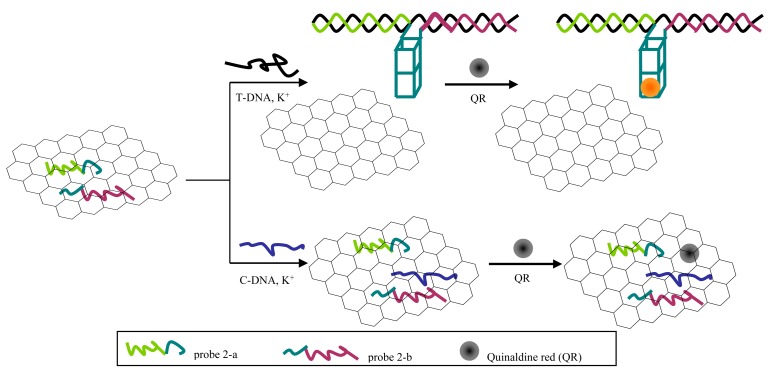
Principle of the split G-quadruplex/GO platform for fluorescence identifying PH.

Scheme 1 shows principle of the split G-quadruplex/GO based fluorescence platform for discriminating PH from its counterfeit species.

In the absence of T-DNA, probes in single-stranded conformation can be adsorbed on the GO surface and even C-DNA added can barely release the probes. In the presence of T-DNA, the probes hybridize with T-DNA to form rigid double-stranded structures. However, this double-stranded DNA cannot be stably adsorbed on the GO surface due to its negatively charged phosphate backbone. Furthermore, in the presence of K^+^, the overhanging G-rich sequence mutually combines to shape a G-quadruplex structure which helps the compound to keep away from the GO platform. Eventually, quinaldine red combines with G-quadruplex specifically and then releases a strong fluorescence signal.

### 3.4. Fluorescence Recovery

**Figure 3.**
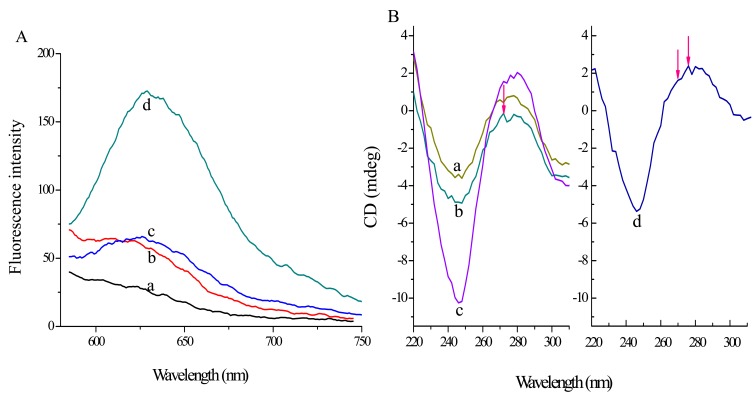
(**A**) Fluorescence spectra of (a) 10^−5^ mol·L^−1^ quinaldine red; (b) 10 mg·L^−1^ GO + 10^−5^ mol·L^−1^ quinaldine red; (c) 10 mg·L^−1^ GO + 10^−7^ mol·L^−1^ probe 2 + 10^−5^ mol·L^−1^ quinaldine red; (d) 10 mg·L^−1^ GO + 10^−7^ mol·L^−1^ probe 2 + 3 × 10^−7^ mol·L^−1^ T-DNA + 10^−5^ mol·L^−1^ quinaldine red; (**B**) CD spectra of (a) probe 2; (b) GO + probe 2; (c) GO + probe 2 + T-DNA; (d) CD spectrum of subtracting curve b from c.

To further demonstrate the principle, fluorescence recovery of the split G-quadruplex/GO platform has been studied. As shown in Figure 3A, fluorescence signal of quinaldine red itself (curve a) is quite low and then increases a little as 10 mg·L^−1^ GO is added (curve b).

Moreover, the signal has nearly no change after incubation with probe 2 (curve c, *F*_0_), which indicates that the probes are completely adsorbed on the GO surface. Furthermore, as 3 × 10^−7^ mol·L^−1^ T-DNA is added, the fluorescence signal (*F*, curve d) increases dramatically with fluorescence recovery efficiency (*F/F*_0_) of 261.8%. Fluorescence recovery efficiency is calculated by *F/F*_0_, where *F*_0_ and *F* represent fluorescence intensity of the split G-quadruplex/GO system in the absence and presence of T-DNA, respectively. The result demonstrates that the probes can hybridize with T-DNA and the overhanging G-rich sequence shapes G-quadruplex structure combine with quinaldine red to release a strong fluorescence signal.

Meanwhile, CD and a subtractive method are also used to verify the principle. According to reports, the CD spectrum of a typical parallel G-quadruplex structure has a positive peak near 270 nm and a negative band around 240 nm, while a positive peak at 295 nm and a negative peak near 265 nm can be characterized in typical antiparallel G-quadruplex structures [27]. In addition, the CD spectrum of a B-form duplex DNA structure possesses a positive peak around 277 nm and a negative band close to 245 nm [28]. As shown in Figure 3B, the CD signal of curve b shifts negatively compared to that of curve a, which indicates the G-quadruplex structure can be affected by the GO platform. Furthermore, the influence of T-DNA can be determined by curve d by subtracting curve b from c. We can find that curve d has a positive peak around 277 nm, a shoulder peak at 270 nm and a negative band near 245 nm. The result suggests that the probes combine T-DNA to construct a B-form duplex and the overhanging G-rich repeats can shape a parallel G-quadruplex structure.

### 3.5. Optimization of Factors

**Figure 4.**
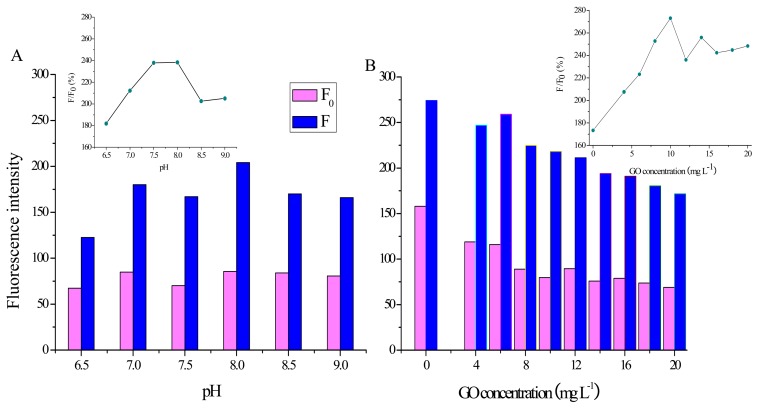
Fluorescence intensity of the GO/probe 2/quinaldine red platform. (**A**) in pH from 6.5 to 9.0 respectively in the absence of T-DNA (*F*_0_, colored magenta) or presence of T-DNA (*F*, colored blue). Inset: *F*/*F*_0_ in pH from 6.5 to 9.0; (**B**) in GO from 0 to 20 mg·L^−1^ respectively in the absence of T-DNA (*F*_0_, colored magenta) or presence of T-DNA (*F*, colored blue). Inset: *F/F*_0_ in GO from 0 to 20 mg·L^−1^.

A series of working buffers with pH values from 6.5 to 9.0 have been prepared and their effect on the fluorescence recovery efficiency has been investigated. As shown in Figure 4A, while *F*_0_ has nearly no change with the increasing pH, the value of *F* increases greatly from 6.5 to 7.0 and then possesses a maximum at pH 8.0. Moreover, *F/F*_0_ increases with pH from 6.5 to 7.5, followed by a stationary phase from 7.5 to 8.0 and then a decrease after 8.0 (inset in Figure 4A). Therefore, pH 8.0 is used in the following experiments.

The influence of GO concentration (from 0 to 20 mg·L^−1^) on the recovery efficiency has also been studied. As shown in Figure 4B, while both *F*_0_ and *F* decline gradually with the increase of GO concentration, *F/F*_0_ increases rapidly with the GO concentration from 0 to 10 mg·L^−1^ and then shows little change after 12 mg·L^−1^ (inset in Figure 4B). From the results, we can conclude that 10 mg·L^−1^ GO nanosheets provide enough space and suitable force for adsorbing probes. Therefore, 10 mg·L^−1^ GO dispersion is used in the following experiments.

### 3.6. Analysis of T-DNA and Specificity Study

**Figure 5.**
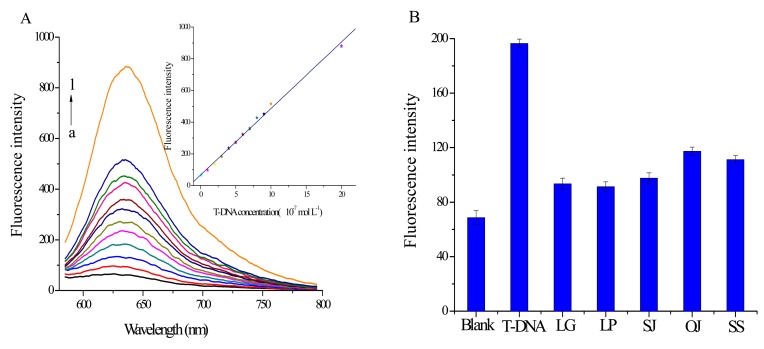
Fluorescence spectra of the split G-quadruplex/GO platform. (**A**) with T-DNA (a) 1.0 × 10^−8^; (b) 1.0 × 10^−7^; (c) 2.0 × 10^−7^; (d) 3.0 × 10^−7^; (e) 4.0 × 10^−7^; (f) 5.0 × 10^−7^; (g) 6.0 × 10^−7^; (h) 7.0 × 10^−7^; (i) 8.0 × 10^−7^; (j) 9.0 × 10^−7^; (k) 1.0 × 10^−6^; (l) 2.0 × 10^−6^ mol·L^−1^. Inset: derived calibration curve of the fluorescence intensity at 635 nm *vs.* T-DNA concentration; (**B**) in the absence of T-DNA or presence of T-DNA (3.0 × 10^−7^ mol·L^−1^) or C-DNA (3.0 × 10^−7^ mol·L^−1^).

The relationship between the fluorescence signal and T-DNA concentration is investigated under the optimal experimental conditions. Figure 5A indicates that the fluorescence signal increases continuously with T-DNA concentration from 1.0 × 10^−8^ to 2.0 × 10^−6^ mol·L^−1^. Furthermore, the inset in Figure 5A is the calibration curve for T-DNA detection. It shows the fluorescence intensity is linearly dependent on the T-DNA concentration in the range from 1.0 × 10^−8^ to 2.0 × 10^−6^ mol·L^−1^ (coefficient *R*^2^ = 0.9974). The detection limit is estimated to be 7.8 × 10^−9^ mol·L^−1^ (3*S*_0_/*S, S*_0_ is the standard deviation for the blank solution, *n* = 7; *S* is the slope of the calibration curve). These results clearly demonstrate that the split G-quadruplex/GO platform can be a sensitive approach for identifying T-DNA.

In order to discuss the specificity of this split G-quadruplex/GO platform, control experiments with the main counterfeit species C-DNA with the same concentration of T-DNA have been done. As shown in Figure 5B, the fluorescence signal exhibits a much lower response to C-DNA than T-DNA, which demonstrates the binding force between C-DNA and probes is too weak to drive probes away from GO surface. Therefore we can conclude that the split G-quadruplex/GO based sensor can be utilized for authentication of PH from its main counterfeit species with little interference.

## 4. Conclusions

In the present study, we have developed a sensitive and selective platform for distinguishing and authenticating PH from its main counterfeit species using the nrDNA ITS sequence as DNA marker. In the platform, 2:2 split G-quadruplex probes are designed and GO nanomaterial is first introduced to reduce their background signal. Furthermore, in the presence of T-DNA, the probes hybridize with T-DNA for double-stranded structures and their overhanging GGG repeats assembles into a G-quadruplex structure. The complex moves away from the GO surface and combines with quinaldine red to release a strong fluorescence signal. Importantly, the probes are label-free and the whole experimental process is fast and simple. Moreover, the background of probes is significantly reduced. We hope this work may be combined with the PCR technique and serve as a foundation of further development of G-quadruplex/GO sensors for the authentication of Traditional Chinese Medicines or other areas.

## Figures and Tables

**Table 1.** Oligomers used in the fluorescence test of quinaldine red.

**Oligomer**		**Sequence (from 5′ to 3′)**
1	GC	(GC)_6_
2	LD	GCGCA_2_T_2_GCGC
3	LQ1	TG_4_T
4	Hum12	(T_2_AG_3_)_2_
5	Apt	G_2_T_2_G_2_TGTG_2_T_2_G_2_
6	PS2.M	GTG_3_TAG_3_CG_3_TTG_2_

**Table 2.** Oligomers used in the fabrication of this split G-quadruplex/GO platform for authentication of PH from its counterfeit species.

**Oligomer**		**Sequence (from 5′ to 3′)**
T-DNA	PH	CGAAACCTGCCCAGC----AGAACGACCAGCGAACAT

C-DNA	LG	CGTGACCCTT--AAC----AAAACAGACCGCGCACG
	LP	CAACACGTGTGCAGTTTAGAGCATACTCA ATA AACA
	OJ	CAATACGTGTG-AGTTTA-AGCATACTCA ATA AACA
	SS	CAATACATGTGCAGTTTA-AGCATACTCAGTGAACA
	SJ	CAGTACATGTGCAGTTTAGAGCATA-TCA ATA AACA

probe	probe-1	G_3_TTG_3_CG_3_ATG_3_TAAAACATGTTCGCTGGTCGTTCTGCTGGGCAGGTTTCG
	probe 2-a	G_3_TTG_3_CCGTTCTGCTGGGCAGGTTTCG
	probe 2-b	AGTGTAAAACATGTTCGCTGTG_3_TAG_3_
